# ASSOCIATIONS BETWEEN COGNITIVE FUNCTION AND BIOACTIVE FOOD COMPOUNDS IN 100% WATERMELON JUICE

**DOI:** 10.1093/geroni/igz038.2433

**Published:** 2019-11-08

**Authors:** Amy Ellis, Ann Bradford Hansen, Navneet Baidwan, Vinoth Aryan Nagabooshanam, Kristi Crowe-White

**Affiliations:** 1 The University of Alabama, Tuscaloosa, Alabama, United States; 2 University of Alabama at Birmingham, Birmingham, Alabama, United States

## Abstract

Objectives: Decline in cognitive function and increases in inflammation and oxidative stress are part of normal aging. Watermelon contains numerous bioactive compounds including lycopene, arginine, and citrulline that exhibit both anti-inflammatory and antioxidant functionality. Thus, the objective of this study was to examine the effect of 100% watermelon juice supplementation on cognitive performance. Methods: A placebo-controlled, randomized, double-blind, crossover trial was conducted with postmenopausal women (n = 16, 60 + 4.1y). Participants initiated a low-lycopene diet during a one-week run-in period and adhered to this diet throughout the study. For four weeks, participants were randomized to consume either two 360 mL servings of pasteurized 100% watermelon juice or a placebo beverage. Following a two-week washout period, participants received the opposite beverage for an additional four weeks. Pre/post each intervention arm, fasting blood samples were collected, and cognitive tests were administered to assess various neurocognitive domains. Statistical analyses included mixed models and Spearman correlations. Results: Serum lycopene exhibited a significant treatment effect (p=0.002); however, lycopene was not correlated with any cognitive test. In contrast, no significant treatment effect was observed for serum arginine or citrulline, yet arginine was significantly inversely correlated with Digit Span Forward (p = 0.005, r = -0.547) and Letter Fluency (p = 0.024, r = -0.507). Conclusion: Despite research supporting the relationship between lycopene and enhanced cognition, lycopene was not related to improvements in cognitive performance in this study. Nevertheless, consumption of 100% watermelon juice may be beneficial for increasing circulating levels of this antioxidant.

